# Mechanistic Models of Virus–Bacteria Co-Infections in Humans: A Systematic Review of Methods and Assumptions

**DOI:** 10.3390/pathogens14080830

**Published:** 2025-08-21

**Authors:** Mani Dhakal, Brajendra K. Singh, Rajeev K. Azad

**Affiliations:** 1 Department of Mathematics, University of North Texas, Denton, TX 76203, USA; manidhakal@my.unt.edu; 2 The Preserve at Killian Hill, Lilburn, GA 30047, USA; brajendra98@yahoo.com; 3 Department of Biological Sciences and BioDiscovery Institute, University of North Texas, Denton, TX 76203, USA

**Keywords:** mathematical modeling, mechanistic models, virus–bacteria interactions, co-infection, model assumptions, parameterization, HIV-TB

## Abstract

Background: Viral–bacterial co-infections can amplify disease severity through complex biological mechanisms. Mathematical models are critical tools for understanding these threats, but it is unclear how well they capture the underlying biology. This systematic review addresses a central question: to what extent does the current generation of models mechanistically represent co-infections, or do the mathematical assumptions underlying these models adequately represent the known biological mechanisms? Methods: Following PRISMA guidelines, we systematically reviewed the literature on mechanistic models of human virus–bacteria co-infections. A systematic search of articles on the scientific literature repositories PubMed, Scopus, and Dimensions was conducted and data on study objectives, model structure, assumptions about biological interactions (e.g., susceptibility, mortality), control measures (if evaluated), and the empirical sources used for key parameters were extracted. Results: We identified 72 studies for inclusion in this analysis. The reviewed models are consistently built on the established premise that co-infection alters disease severity and host susceptibility. However, we found they incorporate these dynamics primarily through high-level mathematical shortcuts, such as applying static “multiplicative factors” to transmission or progression rates. Our quantitative analysis also revealed questionable approaches; for example, 79% (57) of these studies relied on non-empirical sources (assumed or borrowed values) for parameter values including interaction parameters (e.g., increased susceptibility to a secondary pathogen following primary infection, or elevated mortality rates in co-infected individuals). Conclusions: An apparently unjustified practice exists in co-infection modeling, where complex biological processes are simplified to fixed numerical assumptions, often without empirical support. This practice limits the predictive reliability of current models. We identify an urgent need for data-driven parameterization and interdisciplinary collaboration to bridge the gap between biological complexity and modeling practice, thereby enhancing the public health relevance of co-infection modeling.

## 1. Introduction

Virus–bacteria co-infections represent a significant and complex public health challenge [1,2]. Historically, major influenza pandemics—including those in 1918, 1957, and 1968—were all linked to secondary bacterial pneumonia as a major cause of mortality [3]. This lethal synergy—where a primary viral infection damages host defenses and predisposes the host to a secondary bacterial invasion—is also a major concern in other pairings, such as human immunodeficiency virus (HIV) with *Mycobacterium tuberculosis* [4,5,6]. The underlying biological interactions between virus and bacteria are complex [7]. For instance, influenza virus-induced damage to the respiratory epithelium facilitates bacterial colonization [8], and HIV-induced immune suppression increases susceptibility to a wide range of bacterial infections [9]. Complicating these dynamics further are phenomena like viral interference, where the presence of one virus can dampen the replication of another [9]—an antagonistic interaction with potentially detrimental effects on other co-circulating viruses, but beneficial for the human host.

Understanding and predicting the dynamics of virus–bacteria co-infections remain challenging due to the complex interactions, and often unknown mechanisms underlying such interactions, between pathogens and hosts. To investigate these complex dynamics, researchers have increasingly turned to mathematical and computational models [2,10,11,12]. These models can integrate mechanistic knowledge, simulate counterfactual scenarios, and probe questions that are difficult to answer using empirical data alone—for example, how one pathogen’s presence affects another’s transmission, or how to optimize interventions targeting both threats concurrently [5]. The urgency of past public health crises, including the HIV–tuberculosis (HIV-TB) syndemic, the 2009 H1N1 influenza season, and the COVID-19 pandemic, has further accelerated the development and application of such models [13,14,15,16].

The approaches used in the modeling literature vary widely, creating a need for a comprehensive synthesis of the field. While several reviews address virus–bacteria co-infections from clinical or microbiological perspectives [1,7,8,10,17,18,19,20], those focused on mathematical modeling remain scarce. As of this writing, only one study [12] has attempted a systematic review with meta-analysis of co-infection models, while another group has published a review protocol [21]. To distinguish our contribution and highlight a key aspect of co-infection modeling, we conduct a focused analysis centered on the following question: how are complex biological interactions that underlie co-infections modeled? We specifically examine population-level mechanistic models to understand how interactions between viruses and bacteria are accounted for within quantitative frameworks.

In conducting this review, we investigated the assumptions underpinning these models. Our analysis reveals a significant disconnect between the known biological complexity of co-infections and the common modeling practices. We found that the field predominantly relies on simple parameter modifications, often without rigorous justifications—such as multipliers for susceptibility or transmission—to capture the impact of co-infection dynamics. This key issue with parameterization, where critical interaction terms often lack empirical support, forms the central critique of our review.

By systematically reviewing the literature from the last three and a half decades, we aimed to discern how these models handle co-infections, what assumptions they make about pathogen interactions, and—most critically—how model parameters are estimated. Our goal is to inform modelers and public health researchers about the current state of co-infection modeling, highlight findings that are consistent (or not) across different studies, and identify key methodological gaps that must be addressed to advance the field.

**Figure 1:**
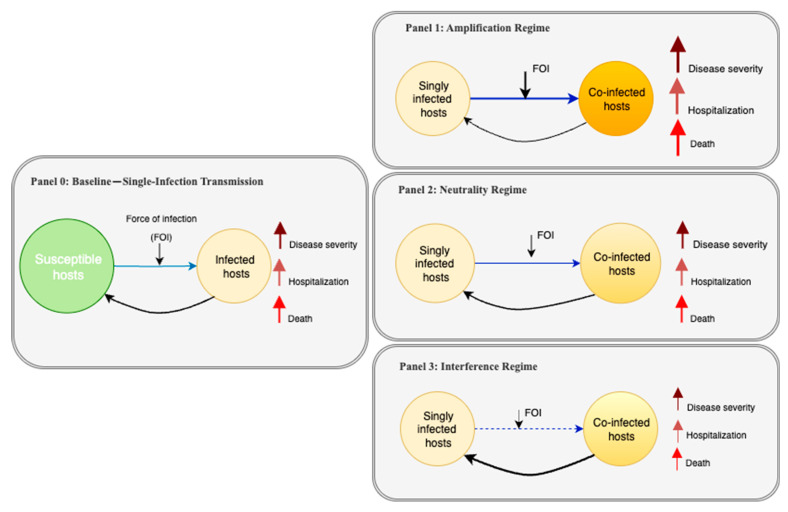
Conceptual models illustrating how co-infection with two pathogens can influence disease outcomes. Panel 0 shows a baseline infection scenario, where susceptible hosts may become infected with either pathogen A or B. Panels 1–3 depict three possible co-infection outcomes: amplification (Panel 1), where co-infection increases severity, hospitalization, and death; neutrality (Panel 2), where co-infection does not alter outcomes [compared to the baseline]; and interference (Panel 3), where co-infection leads to reduced impact. Each panel includes arrows representing severity, hospitalization, and death. While not shown visually, the overall “risk” in each scenario can be interpreted as a composite of these outcomes—higher in amplification, unchanged in neutrality, and lower in interference compared to the baseline.

To visually frame the core concepts and assumptions prevalent in the literature, we introduce a conceptual diagram that illustrates how a baseline single-infection model can be modified to represent different co-infection scenarios (Figure 1). This framework begins with a baseline single-infection transmission model (Panel 0) and then depicts three distinct co-infection modifications or “regimes”: an amplification regime where outcomes are synergistic (Panel 1), a neutrality regime where they are additive (Panel 2), and an interference regime where they are antagonistic (Panel 3). Throughout this review, we will use this framework to discuss how the models we analyzed implement these different regimes and critique the empirical basis for their assumptions.

## 2. Methods

We followed the Preferred Reporting Items for Systematic Reviews and Meta-Analyses (PRISMA) guidelines [22] for this review. A systematic search of electronic databases (PubMed, Scopus, and Dimensions) was conducted to identify articles published up to April 2025. Search terms contained synonyms, or combinations thereof, related to viral infections (e.g., “virus”), bacterial infections (e.g., “bacteria”), co-infections, and mathematical modeling (e.g., “mathematical model,” “simulation,” “dynamics”). To ensure a comprehensive search, we also screened the reference lists of included articles and used Google Scholar to identify any additional relevant literature. The search was conducted for articles published from January 1990 to April 2025 to capture the modern era of computational modeling, though the earliest study that met our inclusion criteria was published in 1997, and was limited to articles in English due to resource limitations for translation.

**Figure 2:**
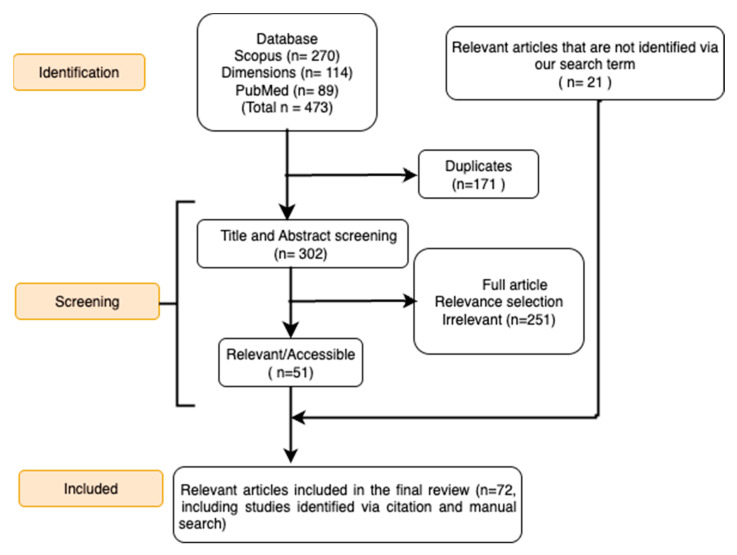
Flow diagram showing the steps involved in the PRISMA process. This diagram illustrates the process of identifying and selecting relevant studies for the systematic review, following the PRISMA guidelines.

Following deduplication, records were screened by title and abstract to exclude irrelevant studies. Eligible articles were those presenting a mechanistic mathematical model of at least one viral and one bacterial pathogen that interact within a human population. For the purpose of this review, a “mechanistic model” was operationally defined as any mathematical model that explicitly represents the transmission dynamics between individuals or populations through a set of equations (e.g., differential equations, agent-based rules) [23]. These models must include compartments or states representing distinct stages of infection (e.g., susceptible, infected, co-infected) and define the transitions between them based on proposed biological or epidemiological mechanisms. This included studies that used deterministic or stochastic compartmental models with explicit transmission dynamics. Additionally, we included within-host models that mechanistically describe the interactions between viruses and bacteria, as these provide foundational insights into the assumptions used in larger-scale models. We excluded purely statistical studies, clinical reports without original modeling, review articles, book chapters, and other non-peer-reviewed works. The full texts of potentially eligible articles were then assessed. Figure 2 displays the PRISMA flow diagram outlining the study selection process.

Key data were extracted from each included study using a standardized form designed to answer our central research question. We collected bibliographic information (authors, year, journal), study objectives, and disease pairs modeled. To specifically investigate how a co-infection is represented and modeled, we extracted detailed data on (i) model structure (e.g., compartmental type, number of compartments) and key simplifying assumptions (e.g., homogeneous mixing); (ii) the specific mathematical methods used to represent co-infection interactions (e.g., parameter multipliers, modified rates); and (iii) the source (empirical or assumed) and justification for all key parameters. These extracted characteristics were then systematically used to categorize the studies, analyze trends, and synthesize the findings presented in the Results section. Data synthesis involved both a quantitative summary of study characteristics and a narrative synthesis of the modeling approaches and their underlying assumptions. This allowed us to first map the landscape of the literature and then perform a detailed analysis of the parameterization practices across the field.

Furthermore, to quantify the focus of the existing literature, we also categorized each included study according to the primary interaction regime it modeled, as depicted in our conceptual framework (Figure 1). As part of this analysis, for studies relying on non-empirical parameters, we further categorized the most frequently assumed interaction terms (e.g., susceptibility multipliers, co-infection mortality) and tabulated their assumed value ranges to quantify trends in modeling assumptions.

## 3. Results

Our systematic search and selection process identified 72 studies for inclusion in this analysis (Figure 2).

### 3.1. An Overview of the Co-Infection Modeling Landscape

Table 1 shows the categories of the 72 included studies based on their primary modeling objectives. Most studies (42 out of 72) focused on evaluating control strategies and interventions, highlighting the central role of models in guiding the public health response to co-infections. Twenty-eight studies aimed to develop models that assessed co-infection dynamics and their impact on host populations, while ten studies examined the influence of specific factors. A smaller number examined within-host or immune system processes (two studies) or focused on forecasting objectives (four studies). This distribution reflects the field’s diverse research interests, with a strong emphasis on understanding the interplay of transmission dynamics and control. Many studies spanned multiple categories, so the total of studies listed across categories exceeds the number of unique studies (72).

**Figure 3:**
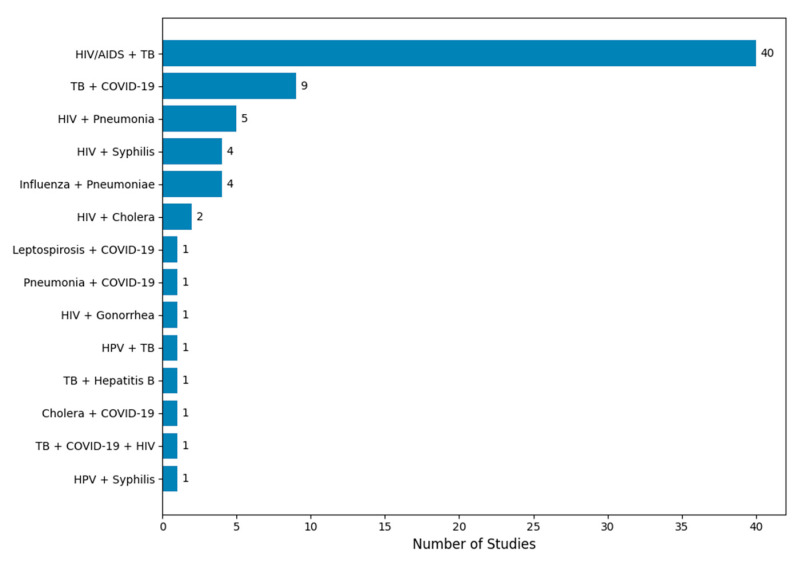
Distribution of co-infection modeling studies by disease pair. The figure displays the number of mechanistic modeling studies categorized by the disease pair modeled. HIV/AIDS and tuberculosis (TB) was the most frequently studied co-infection pair, appearing in 40 out of 72 studies. Other commonly modeled pairs include TB + COVID-19, HIV + pneumonia, and influenza + secondary bacterial pneumonia. Less frequently studied combinations include HIV + cholera, HPV + TB, and triple co-infections such as TB + COVID-19 + HIV. The frequency distribution highlights the research focus on high-burden co-infections and the relative scarcity of models addressing emerging or neglected disease interactions.

The vast majority of included studies modeled two-pathogen interactions, with only a few analyzing more than two pathogens (Table 2). The disease pairs modeled in these studies included HIV, TB, syphilis, pneumonia, COVID-19, cholera, influenza, Human Papillomavirus (HPV), Leptospirosis, Gonorrhea, and Hepatitis B. The distribution of these modeled pairs is visualized in Figure 3. The most frequently modeled pairs were HIV-TB (40 studies), followed by TB-COVID-19 (9 studies), HIV–pneumonia (5 studies), and HIV–syphilis (4 studies). Notably, 65 of these studies involved either HIV or TB, underscoring their dominance in the co-infection modeling literature.

### 3.2. What Shared Patterns and Assumptions Emerge from the Reviewed Co-Infection Modeling Studies?

**Figure 4:**
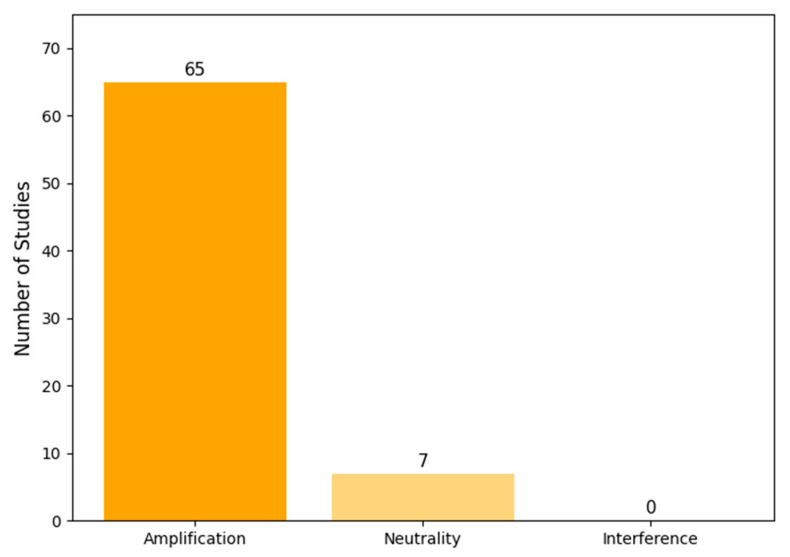
Distribution of modeled interaction regimes across the 72 reviewed studies. The vast majority of models focused on amplification (synergistic) effects, with very few modeling neutralities and none modeling interference (antagonistic) effects.

Our classification based on the conceptual framework (Figure 1) revealed that the vast majority of studies focused on synergistic interactions (Figure 4). Of the 72 included models, 65 (90%) exclusively modeled an amplification regime, where co-infection leads to worse outcomes. A smaller number, seven (10%), modeled a neutrality regime, often as a baseline for comparison. Notably, no models explicitly represented an interference regime where one pathogen could inhibit the other.

These reviewed studies—most of which featured amplification regimes—consistently agree that virus–bacteria co-infection significantly worsens disease outcomes compared to single-pathogen infections. A common point across many studies is that a primary infection with one pathogen often increases host susceptibility to the second pathogen, at least temporarily, a dynamic represented by the transition from a singly infected to a co-infected state in our framework (Figure 1). Furthermore, several modeling studies indicated that co-infections could accelerate the progression of single or all co-occurring diseases, such as the faster progression from HIV to acquired immunodeficiency syndrome (AIDS) in the presence of TB [38,69].

Intervention analyses, a major focus of many studies (Table 3), consistently highlighted the importance and effectiveness of control measures, such as treatment and vaccination. Notably, strategies combining interventions targeting both pathogens, such as concurrent prevention efforts and case finding/treatment, were frequently identified as the most effective or cost-effective approaches to reduce disease burden and transmission [40,46,66,68,84]. The importance of early treatment [24], the role of behavior modification [65], and the constraints imposed by healthcare system capacity were also highlighted as significant factors influencing co-infection dynamics and control [52,96].

From a mathematical standpoint, the models employed standard analytical techniques. The basic reproduction number (*R*_0_) for the co-infection system was typically determined by the maximum of the reproduction numbers of the individual diseases. Correspondingly, these models generally featured a stable, disease-free state when this governing *R*_0_ was less than one, providing a clear threshold for control.

However, not all model outcomes assume synergy [46,64,71]. Some models are designed to represent neutral or even antagonistic interactions, corresponding to the neutrality regime (Panel 2) and interference regime (Panel 3), respectively [97,98]. This is a critical point, as these exceptions help define the boundaries of the synergy assumption. For example, in analyzing influenza and pneumococcus dynamics, Shrestha et al. found that while the virus dramatically increased susceptibility to and severity of bacterial pneumonia, the co-infection had little effect on the independent transmission efficiency (the basic reproduction number) of influenza itself [99,100]. This finding suggests that the interaction is not universally synergistic but is specific to certain biological pathways (host susceptibility vs. pathogen transmissibility). Such counterexamples are vital because they challenge the default assumption of universal amplification and force a more nuanced, mechanism-specific approach to modeling. In summary, while the prevailing conclusion across studies is that viral–bacterial co-infection tends to exacerbate disease (through higher infection risk or mortality), the magnitude and duration of this effect are highly dependent on the specific biological interactions being modeled.

### 3.3. How Do the Models Incorporate Co-Infection Dynamics into Mathematical Models?

Model structures: Most studies used compartmental modeling frameworks such as the classic Susceptible–Infected–Recovered framework, the Susceptible–Exposed–Infected–Recovered framework, and their extensions, with ordinary differential equations governing the flow of individuals between states. In addition, some studies utilized advanced mathematical/modeling techniques, such as partial differential equations [26], fractional derivatives [43,44,45,61,73,85,92], Bayesian inferences [93], data-driven methods [101], stochastic models [61], or agent-based models [77,95]. The models vary significantly in structure and the number of compartments, even when considering the same disease pairs. This variation is influenced by the complexity of the co-infection dynamics being modeled and the specific objectives of the study. Typically, the reviewed models have 6-14 compartments, with a few using 15–24 or more to account for additional disease stages, treatment states, or demographic factors. Critically, every model explicitly includes compartments for co-infected individuals, establishing a common framework for linking two single-pathogen systems.

The reviewed models incorporate co-infection dynamics through several common assumptions about pathogen interactions. Many models allow for superinfection, where a host in a viral-infected class can move to a co-infected class upon bacterial exposure, and vice versa (e.g., [46,51]). The infectiousness of these co-infected hosts is generally modeled as the sum or product of the individual pathogen contributions, sometimes with an additional “co-infection factor” [49,66,83]. For example, a couple of influenza–pneumococcus models ([50,95]) implement an amplification regime (Figure 1, Panel 1) by assuming that influenza infection increases the rate of acquiring pneumococcus, often by a fixed multiplicative factor applied to the force of infection (FOI). Assumptions about mortality and recovery from co-infected states also vary; most studies (e.g., [42,81]) include an extra death rate to represent synergy, while a few (e.g., [39,84]) assume additive mortality (summing the rates from each pathogen) [42,81]. Some sophisticated models [26,41,48,67,82] include multiple disease stages or age structures, but the core idea of linking two single-pathogen models via the co-infection factors is common. A key finding of our review is that justification or the quantitative validation for such parameter adjustments is often lacking in the texts.

Further, to validate the calculated analytical results or to simulate different co-infection scenarios, all the included studies have performed numerical studies. These studies include the calculation of sensitivity indices to identify the most sensitive parameters involved in the model. This was most often performed using the “Forward-Normalized Sensitivity Method,” indexing sensitivity to the basic reproduction number. However, a few studies have also used the Latin hypercube sampling–partial rank correlation coefficient method [102] to identify the influence of each parameter on the calculated reproduction number. These numerical simulations played a crucial role in exploring complex co-infection dynamics and investigating specific scenarios.

### 3.4. Parameterization Practices in Co-Infection Models: Data Sources, Model Fitting, and Validation

**Figure 5:**
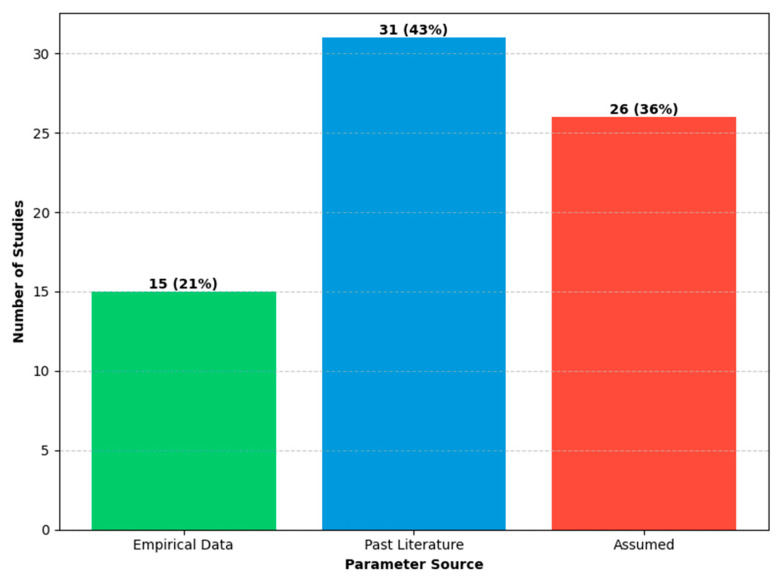
Primary parameter sources used in the reviewed co-infection models. The bars represent the numbers and percentages of studies that relied primarily on empirical data (15 studies, 21%), the past literature (31 studies, 43%), or assumed values (26 studies, 36%). For this classification, studies were assigned to the “Empirical Data” category if they used any real-world data to inform their parameters; the remaining studies were then classified based on their majority source.

Data sources: Model parameterization in the reviewed literature draws on varied and often sparse data sources. Our quantitative analysis, summarized in Figure 5, reveals that 57 out of the 72 studies based their analysis primarily on parameter values that were either assumed or borrowed directly from the previous literature. Only a small minority (15 studies, or 21%) utilized any real-world empirical data for parameterization. Furthermore, even in these cases, the data were often used for partial calibration of single-disease or demographic parameters, not for the crucial co-infection interaction terms themselves. Parameters intended to capture the specific effects of co-infection—such as increased susceptibility, altered transmission, and divergent outcomes visually depicted in Figure 1—almost universally lacked direct empirical grounding and transparent justification.

Fitting and validation: Consistent with the findings on parameter sources, only a minority of models were explicitly fitted to epidemiological data. For example, Shrestha et al. fitted a transmission model to 20 years of state-level flu and pneumonia data to disentangle hypotheses [99]. Most models, however, used plausible parameter ranges from the literature or expert opinion, and validation was generally qualitative (e.g., checking that model outputs resemble observed epidemic curves). Sensitivity or uncertainty analysis was standard: many studies reported how model conclusions change if interaction rates or other key parameters are varied.

### 3.5. Analysis of Non-Empirical Interaction Parameters

Our detailed analysis of the studies that relied on non-empirical parameters revealed several trends in how co-infection dynamics are modeled. The most common parameters used to model the amplification regime were multipliers for increased susceptibility (or FOI) to a secondary pathogen and additional mortality due to co-infection. Table 4 summarizes the distribution of assumed values for these key parameters.

The analysis showed that the assumed multiplicative increase in susceptibility typically ranged from 1 to 3.6. Similarly, the additional mortality rate for co-infected individuals was often modeled by introducing a distinct parameter with a distinct value while very few applied simple additive or multiplicative factors to the baseline mortality rate of the corresponding single infection. Importantly, the parameter values or multiplicative factors chosen were not consistent within studies and no justification was provided. This practice highlights a critical gap: while modelers correctly identify the mechanisms to modify (susceptibility, mortality), the magnitude of these modifications is rarely informed by empirical evidence, relying instead on assumption or values borrowed from other modeling studies that were themselves based on assumptions.

## 4. Discussion

Our systematic review of virus–bacteria co-infection modeling studies reveals a significant gap between the known biological complexity of these interactions and the common practices used to represent them mathematically. While the reviewed studies consistently incorporate the view that co-infection amplifies disease burden, this consensus assertion apparently rests on a weak empirical foundation. The complex biological processes known to drive co-infections are overwhelmingly represented by simplified mathematical assumptions (e.g., fixed susceptibility or transmission multipliers), rather than modeled mechanistically. This gap, driven by a lack of empirical data for key interaction parameters, fundamentally limits the reliability of current models for quantitative prediction and public health guidance.

### 4.1. Principal Finding: A Disconnect Between Biological Mechanisms and Model Parameterization

The central finding of our review is the widespread reliance on non-empirical parameters to represent key co-infection dynamics. This critique is supported by our quantitative finding that 79% of reviewed studies relied primarily on non-empirical parameters sourced from the previous literature or pure assumption (Figure 5). The parameters governing the specific effects of co-infections—such as the use of a multiplicative factor for increased susceptibility—almost universally lacked direct empirical grounding. For example, numerous models of HIV-TB or influenza–pneumonia correctly assume that one infection increases susceptibility to the other (amplification regime, Figure 1, Panel 1), but they accounted for this by adjusting a transmission parameter by a fixed factor, without any data to support the magnitude of that adjustment. Justification for these values is consistently absent, a practice that hinders both reproducibility and context-specific validation.

The consequences of this parameterization gap are twofold. First, it impedes robust model validation. It is inherently difficult to assess a model’s predictive accuracy when its most critical interaction terms are not based on evidence. Second, it hinders the exploration of more complex temporal dynamics known to be important (see Figure 1 in Mochan and Sego [2]), such as whether the sequence of infections alters disease severity, a factor rarely incorporated, possibly due to the intensive data requirements for fitting such models.

### 4.2. Explaining the Gaps: Research Biases and Modeling Challenges

Our analysis reveals a significant bias in the literature toward modeling synergistic (amplification) regimes, while largely overlooking interference mechanisms (Figure 1 and Figure 4). This research focus is understandable, given that the most severe public health crises, such as the high mortality during the 1918 influenza pandemic, are attributed to pathogen synergy, thus directing research priorities toward this “worst-case” scenario. This focus is further compounded by the inherent difficulty and cost of designing experiments or cohort studies capable of precisely measuring interaction parameters. The lack of readily available empirical data creates a cycle where modelers are compelled to borrow or assume parameters, perpetuating the simplifications we observed. The absence of interference modeling represents a major gap, as understanding these dynamics is critical for accurately forecasting seasonal epidemics and interpreting the outcomes of vaccination campaigns that target only one of a pair of competing pathogens.

### 4.3. Strengths and Consistent Findings in the Literature

Despite these limitations, the reviewed models provide significant value. They serve as powerful conceptual tools that consistently highlight the heightened threat posed by co-infections and affirm the need for integrated control strategies. Several reviewed models underscored the potential persistence of co-infections even with certain control thresholds but also affirmed the significant impact of interventions, such as early and continuous treatment [38,69], effective TB control [48], and adequate healthcare system capacity in mitigating co-infection dynamics [89]. These findings reinforce the importance of addressing co-infections while also underscoring the need for more reliable, data-grounded models to accurately quantify these effects and guide interventions.

### 4.4. Limitations of This Review

While this review provides a comprehensive synthesis of the literature, it is important to acknowledge its limitations. First, the search result only found studies published between 1 January 1990, and 5 April 2025. This may have excluded earlier foundational work or recent studies published after the cut-off date. Second, it only considered studies published in English, which may also leave out important studies published in other languages. Third, due to the narrative synthesis approach, a formal meta-analysis of model parameters was not performed, which limits a quantitative comparison of outcomes across studies. Finally, while our search was systematic, the exclusion of non-English literature and the specific keywords and databases chosen may have omitted some relevant work. These limitations underscore the need for caution in generalizing the results of this review.

## 5. Conclusions and Future Directions

In summary, mathematical models have been invaluable tools for conceptualizing and exploring the potential impacts of co-infections. However, for the field to advance from theoretical exploration toward practical public health application, we recommend the following concrete steps. First, modelers must prioritize data-driven parameterization, moving away from assumed interaction multipliers [103]. This requires forming interdisciplinary teams where experimentalists and clinicians can provide context-specific data (e.g., from co-culture experiments, cohort studies) to inform parameter values [104]. Second, future models should aim to incorporate dynamic, rather than static, interactions [105]. For example, instead of a fixed susceptibility factor, models could represent immune suppression as a variable that changes over the course of the primary infection. Finally, there is a critical need to explore under-studied interaction regimes, particularly pathogen interference, as these dynamics have significant implications for herd immunity and multi-pathogen vaccine strategies [106]. By embracing these more rigorous, data-grounded approaches, co-infection modeling can become a more reliable and essential tool for public health decision-making.

## Figures and Tables

**Table 1:** Primary study objectives of the reviewed studies.

Study Objectives	Studies
Modeling novel disease pairs to assess co-infection dynamics and their impact	[24,25,26,27,28,29,30,31,32,33,34,35,36,37,38,39,40,41,42,43,44,45,46,47,48,49,50,51]
Performing intervention analysis and identifying optimal control measures	[24,29,38,40,43,46,48,49,50,52,53,54,55,56,57,58,59,60,61,62,63,64,65,66,67,68,69,70,71,72,73,74,75,76,77,78,79,80,81,82,83,84]
Assessing the impact of specific factors	[25,30,34,52,61,65,81,85,86,87]
Exploring within-host and immune system dynamics	[33,88]
Forecasting or predicting future disease incidence and outcomes	[50,89,90,91]

Note: The total number of studies across categories exceeds 72 as many studies addressed multiple objectives.

**Table 2:** Summary of co-infection disease pairs and model structures in the reviewed literature.

Disease Pair Modeled	Number of Compartments	Number of Studies	References
HIV/AIDS + TB	4–21 (majority ranging in between 8 and 14 with few exceptions)	40	[25,26,28,29,30,31,32,33,34,39,40,41,42,43,44,45,53,54,55,56,61,62,63,65,66,68,72,75,76,78,79,80,82,83,84,87,89,92,93,94]
TB + COVID-19	4–10	9	[35,36,37,59,60,71,86,88,91]
HIV + Pneumonia	9–12	5	[38,69,70,74,85]
HIV + Syphilis	4–15	4	[24,49,67,77]
Influenza + Pneumoniae (or other secondary bacteria)	8–11	4	[50,57,81,95]
HIV + Cholera	5, 6	2	[27,64]
Leptospirosis + COVID-19	9 (host) + 2 (vector)	1	[51]
Pneumonia + COVID-19	5	1	[46]
HIV + Gonorrhea	8	1	[90]
HPV + TB	24	1	[48]
TB + Hepatitis B	13	1	[47]
Cholera + COVID-19	14 (host) and 1 (bacteria)	1	[58]
TB + COVID-19 + HIV	15	1	[52]
HPV + Syphilis	11	1	[73]

**Table 3:** Summary of control measures evaluated in the reviewed co-infection models.

Disease Pair	Studies	Control Measures
HIV/AIDS + TB	[29,40,52,53,55,56,62,63,65,66,68,72,75,78,80,82,84]	prevention efforts, case findings, treatments, awareness campaigns, behavior modifications
COVID-19 + TB	[37,59,60,71]	prevention efforts, better treatment, vaccination, quarantine
HIV/AIDS + Pneumonia	[38,70]	vaccination, treatment
Influenza + Pneumonia	[57,81]	social distancing, vaccination, antiviral resistance management
COVID-19 + Pneumonia	[46]	vaccination, quarantine
HIV + Cholera	[64]	cholera prevention control (vaccination), HIV prevention control, immunity control
COVID-19 + Cholera	[58]	social distancing, quarantine and isolations, distribution of COVID-19 test kits, distribution of chlorine water tablets
HPV + TB	[48]	treatment, condom use
HIV + Syphilis	[67]	syphilis treatment

**Table 4:** Summary of the most common non-empirical parameters.

Parameters Representing Interactions	Common Modeling Approach	Range of Assumed Values *	Number of Studies Using this Approach
Altered Transmission	Multiplicative factor (σ) applied to FOI	σ ∈ [1, 3.6]; some assuming higher values like 7.59 and 106.45	52 (72%)
Altered Mortality	Distinct death rate (δ) for co-infected class	δ ∈ [0.001, 0.75]	58 (81%)
Altered Infectiousness	Multiplicative factor (ε) for co-infected class	ε ∈ [0.02, 3.5]	41 (57%)
Altered Disease Progression	Distinct parameter and their values on progression from different compartments	Varied	56 (78%)

* Ranges are illustrative and represent the typical values observed in the reviewed literature where specific assumptions were stated.

## Data Availability

All datasets/materials are provided with the article. We also declare that no biomolecular data (e.g., proteomics data and protein sequences, DNA and RNA sequences, genetic polymorphisms, linked genotype and phenotype data, macromolecular structure, gene expression data, crystallographic data for small molecules) were generated from this work.
